# Plerixafor combined with standard regimens for hematopoietic stem cell mobilization in pediatric patients with solid tumors eligible for autologous transplants: two-arm phase I/II study (MOZAIC)

**DOI:** 10.1038/s41409-020-0836-2

**Published:** 2020-03-03

**Authors:** Bruce Morland, Tomas Kepak, Sandro Dallorso, Julian Sevilla, Dermot Murphy, Roberto Luksch, Isaac Yaniv, Peter Bader, Jochen Rößler, Gianni Bisogno, Britta Maecker-Kolhoff, Peter Lang, C. Michel Zwaan, David Sumerauer, Gergely Kriván, John Bernard, Qianying Liu, Eileen Doyle, Franco Locatelli

**Affiliations:** 1 Birmingham Women’s and Children’s Hospital, Birmingham, UK; 2 University Hospital Brno and ICRC/St. Anna University Hospital, Masaryk University, Brno, Czech Republic; 3 IRCCS Giannina Gaslini, Genova, Italy; 4 Hospital Infantil Universitario Niño Jesus, FIB HIUNJ, CIBERER, Madrid, Spain; 5 Royal Hospital for Children, Glasgow, UK; 6 Fondazione IRCCS Istituto Nazionale dei Tumori, Milan, Italy; 7 Schneider Children’s Medical Center of Israel, Tel Aviv University, Petah Tikva, Israel; 8 Sackler Faculty of Medicine, Tel Aviv University, Tel Aviv, Israel; 9 Universitätsklinikum Frankfurt am Main, Frankfurt, Germany; 10 Division of Pediatric Hematology/Oncology, Department of Pediatrics, Inselspital, Bern University Hospital, University of Bern, Bern, Switzerland; 11 Department for Women’s and Children’s Health, University of Padua, Padua, Italy; 12 Hannover Medical School, Hannover, Germany; 13 University Children’s Hospital, Tübingen, Germany; 14 Erasmus MC-Sophia Children’s Hospital, Rotterdam, Netherlands; 15 Princess Máxima Center, Utrecht, Netherlands; 16 Faculty Hospital Motol, Prague, Czech Republic; 17 United St Istvan and St Laszlo Hospital, Budapest, Hungary; 18 Sanofi Genzyme, Cambridge, MA USA; 19 Sanofi US, Bridgewater, NJ USA; 20 IRCCS Bambino Gesù Children’s Hospital, Rome, Italy; 21 Sapienza University of Rome, Rome, Italy; 22 Present Address: Mustang Bio Inc., Worcester, Massachusetts USA

## Abstract

This study (NCT01288573) investigated plerixafor’s safety and efficacy in children with cancer. Stage 1 investigated the dosage, pharmacokinetics (PK), pharmacodynamics (PD), and safety of plerixafor + standard mobilization (G-CSF ± chemotherapy). The stage 2 primary endpoint was successful mobilization (doubling of peripheral blood CD34+ cell count in the 24 h prior to first apheresis) in patients treated with plerixafor + standard mobilization vs. standard mobilization alone. In stage 1, three patients per age group (2–<6, 6–<12, and 12–<18 years) were treated at each dose level (160, 240, and 320 µg/kg). Based on PK and PD data, the dose proposed for stage 2 was 240 µg/kg (patients 1–<18 years), in which 45 patients were enrolled (30 plerixafor arm, 15 standard arm). Patient demographics and characteristics were well balanced across treatment arms. More patients in the plerixafor arm (24/30, 80%) met the primary endpoint of successful mobilization than in the standard arm (4/14, 28.6%, *p* = 0.0019). Adverse events reported as related to study treatment were mild, and no new safety concerns were identified. Plerixafor + standard G-CSF ± chemotherapy mobilization was generally well tolerated and efficacious when used to mobilize CD34+ cells in pediatric cancer patients.

## Introduction

Plerixafor (Mozobil®, Sanofi, Cambridge, MA, USA) is an antagonist of the 7 transmembrane G protein coupled chemokine (C-X-C motif) receptor 4 (CXCR4) that works by disrupting the interaction of CXCR4 with stromal cell-derived factor-1, resulting in the release of CD34+ stem cells into the circulation [1–4]. In the United States, plerixafor is licensed for use in combination with granulocyte colony stimulating factor (G-CSF) to mobilize hematopoietic stem cells (HSCs) into peripheral blood (PB) for collection and subsequent autologous HSC transplantation (HSCT) in adult patients with non-Hodgkin lymphoma or multiple myeloma (MM). In the European Union, plerixafor is indicated for use in combination with G-CSF to enhance mobilization of HSCs into PB and subsequent autologous HSCT in adults with lymphoma and MM, who are proven to be poor mobilizers.

While the efficacy and safety of plerixafor is well established in adults, limited data for its use in children are available. This study investigated the appropriate dosing, safety, and efficacy of plerixafor when given in combination with G-CSF, in pediatric patients with different types of cancer.

## Methods

This was a phase I–II international, multicenter, randomized, parallel assignment, open-label study of plerixafor in pediatric cancer patients (ClinicalTrials.gov, NCT01288573; EudraCT, 2010-019340-40). The study was conducted in accordance with the Declaration of Helsinki and the International Conference on Harmonization Guidelines for Good Clinical Practice.

**Fig. 1:**
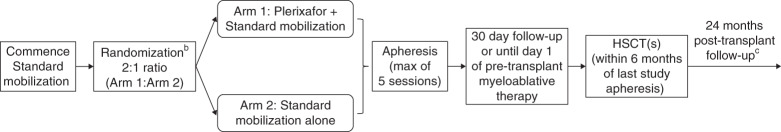
Stage 2 study design. **a** At least 40 patients were to be enrolled into stage 2, which was open to patients with all malignant conditions leading to autologous transplant except leukemia, but with the intention of acquiring a minimum of five patients for each of the following diagnoses: Ewing’s sarcoma/soft tissue sarcoma, lymphoma, neuroblastoma, and brain tumors. **b** Randomization based on day when trigger point of ≥7 CD34+ cells/μL was reached. **c** Twenty-four months after the last planned transplant performed within 6 months or after last study apheresis if not transplanted.

Independent ethics committees or institutional review boards at the participating sites approved the protocol and all amendments. All patients, and/or their legal guardians, provided written informed consent prior to enrollment. The study was conducted in two stages. Stage 1 (3 + 3 design) investigated the appropriate dose, safety, pharmacokinetics (PK) and pharmacodynamics (PD) of plerixafor in pediatric cancer patients when given in addition to standard mobilization (chemotherapy plus daily G-CSF or daily G-CSF alone). Stage 2 was an open-label, comparative study to evaluate the efficacy of plerixafor in addition to standard mobilization regimens of HSCs into PB, and subsequent collection by apheresis in pediatric patients with cancer (Fig. 1).

### Patients

Pediatric patients with Ewing’s sarcoma/soft tissue sarcoma, neuroblastoma, brain tumors, and other malignancies scheduled to undergo high-dose chemotherapy followed by autologous HSCT were eligible for the study (Table 1). For stage 1, patients were aged between 2 and <18 years, and in stage 2, patients were aged 1–<18 years. Full exclusion criteria are provided in the Supplementary Materials.

**Table 1:** Stage 1 patient baseline characteristics.

	Age 2–<6 years (*n* = 9)	Age 6–<12 years (*n* = 9)	Age 12–<18 years (*n* = 9)
Age [years], mean ± SD	3.1 ± (1.2)	8.8 ± 1.7	15.3 ± 1.1
Male, *n* (%)	2 (22)	7 (78)	3 (33)
Race, *n* (%)			
White	8 (89)	9 (100)	8 (89)
Asian	1 (11)	0	1(11)
Ethnicity, *n* (%)			
Hispanic or Latino	0	0	0
Not Hispanic or Latino	7 (78)	9 (100)	8 (89)
Not reported	2 (22)	0	1 (11)
Tumor type, *n* (%)			
Ewing’s sarcoma	0	3 (33)	3 (33)
Hodgkin’s lymphoma	0	0	3 (33)
Medulloblastoma	0	0	0
Neuroblastoma	7 (78)	4 (44)	2 (22)
Non-Hodgkin’s lymphoma	0	1 (11)	0
Rhabdomyosarcoma	1 (11)	0	1 (11)
PNET	1 (11)	0	0
CNS tumor not germinomatous	0	1 (11)	0

### Study procedures

In stages 1 and 2, plerixafor was administered subcutaneously at a separate anatomical site from the patient’s standard mobilization treatment. All participating patients received a standard mobilization regimen (chemotherapy plus daily G-CSF or daily G-CSF alone) as per study site practice guidelines. In stage 1, all patients received plerixafor in addition to the standard mobilization regimen. In stage 2, 45 patients were randomized 2:1 to receive plerixafor plus standard mobilization or standard mobilization alone. In both stage 1 and stage 2, CD34+ cells were obtained from venous blood samples by both local and central laboratories for fluorescence-activated cell sorting (FACS) analysis. CD34+ cell counts were obtained at regular intervals to determine when the trigger point of seven CD34+ cells/µL was reached, at which time plerixafor administration was scheduled for the next day.

In stage 1, three dose levels of plerixafor (160, 240, and 320 µg/kg) were studied for each of the age groups 2–<6 years, 6–<12 years, and 12–<18 years, respectively, (based on the adult dose of 240 µg/kg). Blood samples were taken to determine plerixafor concentrations in the plasma, which were evaluated using liquid chromatography coupled with tandem mass spectrometry with a lower limit of quantification of 5 ng/mL. Venous blood samples were collected for CD34+ FACS analysis on the morning of the day preceding the first apheresis and on the morning of the apheresis day (prior to administration of the daily G-CSF dose). For each apheresis collection, apheresis product volume, absolute number of CD34+ cells per unit volume, and the patient’s weight were used to calculate the yield of CD34+ cells/kg. The total CD34+ cell yield was calculated as the sum of all apheresis collections.

In stage 2, all patients received standard mobilization with G-CSF, dosed at 10 µg/kg daily. In patients mobilizing poorly prior to randomization, the dose of G-CSF could be increased up to a maximum of 15 µg/kg (three (10%) patients in the plerixafor arm and one patient in the standard (6.6%) therapy arm) if this was in accordance with the study site standard practice. Patients were randomized when the trigger point of seven CD34+ cells/µL was reached (a threshold of seven CD34+ cells/µl was chosen as the lowest threshold to maximize the probability that patients could achieve apheresis following randomization).

Plerixafor was dosed at 240 µg/kg (determined in stage 1) between 8 and 12 h before the planned apheresis as CD34+ cell counts peak ~9 h after plerixafor mobilization. A maximum of five apheresis sessions were permitted for each patient. On the morning of the day preceding the first apheresis and on the morning of the apheresis day itself (prior to administration of the daily G-CSF), venous blood samples were taken for PB CD34+ FACS analysis.

The primary stage 2 efficacy endpoint was successful mobilization, defined as at least a doubling of PB CD34+ cell count in the 24 h prior to first apheresis. Secondary stage 2 endpoints included safety, number of days of apheresis required to reach ≥2 × 10^6^ CD34+ cells/kg, CD34+ yield per apheresis, total CD34+ cell yield, and percentage of patients proceeding to transplant, successfully engrafting, and the number of patients with durable engraftment at 3, 6, 12, and 24 months post transplant.

Exploratory endpoints included the CD34+ cell count that was transplanted, increase in CD34+ cell counts, total blood volume processed, time to neutrophil engraftment, and time to platelet engraftment.

Treatment-emergent adverse events (TEAEs) were defined as any adverse event from the date of randomization until 30 days after the last dose of the patient’s study mobilization regimen, or until the first dose of their next anticancer therapy or pretransplant myeloablative therapy, whichever occurred first. Safety was assessed in stage 1 and stage 2 by monitoring TEAEs. All patients who underwent transplant within 6 months of their last study apheresis were evaluated up to 24 months after their last transplant. For patients who did not undergo transplant, safety was monitored up to 24 months after the last dose of study mobilization treatment.

### Statistical assessments

A standard 3 + 3 dose parallel escalation strategy was used to determine dose per age cohort in stage 1. For each planned plerixafor dose (160, 240, and 320 µg/kg), 9 patients (3 per age group [2–<6 years, 6–<12 years, and 12–<18 years]) were to be included for a total of 27 patients. Further three patients would be enrolled in a cohort if a dose limiting toxicity occurred. Escalation would only occur if <2 patients exhibited unacceptable toxicities.

For stage 2, it was planned to enroll 40 patients; 27 patients in the plerixafor arm and 13 in the standard arm (2:1 ratio). The sample size has been determined by the ability to recruit patients to the study, based on known numbers of autologous transplants carried out in pediatric cancer patients, diagnoses to be included in this study, and limiting participation to larger sites that have capability to achieve sufficient patient numbers within a reasonable amount of time. No formal statistics-based on sample size calculations have been performed.

The full analysis set comprised all patients randomized in stage 2. Patients were analyzed according to the treatment arm allocated by randomization (intention-to-treat analysis). Continuous variables were summarized using either mean and standard deviation (SD), or median and range (minimum to maximum). Categorical and ordinal data were summarized using the number and percentage of patients in each treatment arm. All summaries and statistical analyses were generated using SAS software version 9.0 or higher. Test for statistical significance was performed only on the primary efficacy endpoint.

## Results

### Stage 1

Twenty-seven patients were included in three age groups (2–<6, 6–<12, and 12–<18 years) with three patients in each age group treated at each dose level (160, 240, and 320 µg/kg) (Table 1).

Pharmacokinetic parameters are summarized in Table 2. There was considerable variability in PK parameters within each age cohort due to low numbers of patients. As the dose of plerixafor was raised from 160 to 240 µg/kg, exposure (as assessed by *C*_max_) to plerixafor generally increased in all age cohorts; further increases between 240 and 320 µg/kg were observed in the youngest and oldest age cohorts. Mean AUC_0–9 h_ values did not appear to increase between the 240 µg/kg and 320 µg/kg doses in the youngest and middle age groups, but were proportional between the 160 µg/kg and 320 µg/kg doses in the oldest group. There was a trend toward lower exposures in the youngest age categories; however, the trend was not clinically relevant, and exposure was sufficient for a response. In general, at all dose levels, CD34+ cells counts within the first hour after dosing lag behind the maximum plasma concentrations of plerixafor, although there was variation between individual patients (Supplementary Fig. 1a, b). The delayed increase in CD34+ cell count with rising plerixafor concentration is consistent with the PK/PD relationship observed in adults. In addition, the PD response regarding CD34+ cell count was comparable across all dose levels. Therefore, the dose proposed for stage 2 was 240 µg/kg to be given between 8 and 12 h before apheresis. Overall, three patients failed to reach 2 × 10^6^ cells/kg (one patient in the 2–<6 years cohort and two in the 12–18 years cohort (Table 3).

**Table 2:** Stage 1 mean ± SD (geometric mean) [CV%] pharmacokinetic parameters of plerixafor.

	2–<6 years			6–<12 years			12–<18 years		
PK parameter	160 µg/kg (*n* = 3)	240 µg/kg (*n* = 3)	320 µg/kg (*n* = 3)	160 µg/kg (*n* = 3)	240 µg/kg (*n* = 3)	320 µg/kg (*n* = 3)	160 µg/kg (*n* = 3)	240 µg/kg (*n* = 3)	320 µg/kg (*n* = 3)
*C*_max_ (ng/mL)	153 ± 82.1 (137) [53.6]	327 ± 244 (223) [74.6]	465 ± 79.7 (460) [17.2]	288 ± 61.5 (283) [21.4]	625 ± 77.7 (621) [12.4]	604 ± 70.9 (601) [11.7]	496 ± 11.3 (496) [2.3]	784 ± 275 (748) [35.1]	1180 ± 205 (1170) [17.3]
*t*_max_^a^ (h)	1.00 (0.87–1.02)	1.00 (0.62–1.02)	0.63 (0.50–0.67)	1.02 (1.00–1.05)	1.00 (1.00–1.00)	1.02 (1.02–1.08)	0.50 (0.25–0.58)	0.25 (0.25–0.50)	0.52 (0.25–0.52)
*T*_1/2Z_ (h)	1.60, 1.88^b^ (1.73)	2.09, 2.78^b^ (2.41)	1.93 ± 0.468 (1.89) [24.2]	2.35 ± 0.674 (2.29) [28.7]	2.59 ± 0.690 (2.52) [26.7]	2.10 ± 0.392 (2.08) [18.6]	4.04 ± 1.57 (3.89) [39.0]	3.05 ± 0.503 (3.02) [16.5]	3.20 ± 0.598 (3.16) [18.7]
AUC_0–9_ (ng × h/mL)	655, 486^b^ (564)	1750, 1730^b^ (1740)	1520 ± 126 (1510) [8.3]	953 ± 231 (934) [24.2]	2270 ± 475 (2230) [20.9]	2140 ± 401 (2110) [18.7]	1800 ± 363 (1770) [20.2]	2600 ± 556 (2550) [21.4]	4120 ± 100 (4120) [2.4]

^a^Median (min–max).

^b^Individual values (geometric mean) are reported when *n* = 2 due to poorly defined elimination phase in some patients.

**Table 3:** Stage 1 efficacy analysis.

	2–<6 years			6–<12 years				12–<18 years				
Parameter	160 µg/kg (*n* = 3)	240 µg/kg (*n* = 3)	320 µg/kg (*n* = 3)	Overall (*n* = 9)	160 µg/kg (*n* = 3)	240 µg/kg (*n* = 3)	320 µg/kg (*n* = 3)	Overall (*n* = 9)	160 µg/kg (*n* = 3)	240 µg/kg (*n* = 3)	320 µg/kg (*n* = 3)	Overall (*n* = 9)
PB CD34+ cell count doubled, *n* (%)												
Yes	2 (67)	1 (33)	3 (100)	6 (67)	2 (67)	3 (100)	3 (100)	8 (89)	3 (100)	3 (100)	3 (100)	9 (100)
No	1 (33)	1 (33)	0	2 (22)	1 (33)	0	0	1 (11)	0	0	0	0
Missing	0	1 (33)	0	1 (11)	0	0	0	0	0	0	0	0
Patients reaching at least 2 × 10^6^ CD34+ cells/kg, *n* (%)												
Yes	2 (67)	3 (100)	3 (100)	8 (89)	2 (67)	3 (100)	3 (100)	8 (89)	2 (67)	3 (100)	2 (67)	7 (78)
No	1 (33)	0	0	1 (11)	0	0	0	0	1 (33)	0	1 (33)	2 (22)
Missing	0	0	0	0	1 (33)	0	0	1 (11)	0	0	0	0
Number of apheresis to reach 2 × 10^6^ CD34+ cells/kg, *n*/*n* (%)												
1	1/2 (50)	1/3 (33)	2/3 (67)	4/8 (50)	1/2 (50)	3/3 (100)	1/3 (33)	5/8 (63)	2/2 (100)	3/3 (100)	1/2 (50)	6/7 (86)
2	1/2 (50)	2/3 (67)	1/3 (33)	4/8 (50)	1/2 (50)	0	2/3 (67)	3/8 (38)	0	0	1/2 (50)	1/7 (14)
Total CD34+ yield, [cells/kg], median (min, max)	54.63 (1.84, 106.07)	9.62 (7.93, 62.21)	16.54 (2.01, 19.93)	16.54 (1.84, 106.07)	5.84 (4.23, 7.44)	8.85 (4.53, 13.72)	12.72 (3.92, 146.36)	8.15 (3.92, 146.36)	8.94 (0.52, 19.08)	4.95 (3.97, 16.28)	15.14 (1.95, 42.73)	8.94 (0.52, 42.73)

Central laboratory data used, however, when central laboratory value was missing, local laboratory values are used.

No dose limiting toxicities were observed in stage 1. TEAEs were experienced by 16 patients (59%); 9 patients (33%) experienced a serious TEAE. All serious TEAEs were attributed to the effects of mobilizing chemotherapy and not related to study treatment. Four deaths occurred after the 30-day period following the last plerixafor dose; three due to disease progression (none of these patients received HSCT) and one patient through a cerebral hemorrhage, of which treatment with heparin was likely the cause.

### Stage 2

Forty-five patients were enrolled in stage 2 (30 plerixafor arm, 15 standard arm); 35 patients completed all study follow-up visits. Patient demographics and characteristics were well balanced across patient cohorts with little variation in diagnosis of underlying disease between age groups (Table 4). Baseline PB CD34+ cell count (last value recorded prior to randomization) was numerically higher in the standard arm than for the plerixafor arm (27.0 vs. 14.8 cells/μL, respectively; no statistical analysis was carried out on baseline parameters). More patients in the plerixafor arm had PB CD34+ cell counts of <20 cells/µL on the day before first apheresis than in the standard arm (Table 5). Ten patients discontinued the study (Table 6).

**Table 4:** Stage 2 patient baseline characteristics.

	Age group 1–<6 years		Age group 6–<12 years		Age group 12–<18 years	
	Standard arm (*n* = 10)	Plerixafor arm (*n* = 16)	Standard arm (*n* = 3)	Plerixafor arm (*n* = 9)	Standard arm (*n* = 2)	Plerixafor arm (*n* = 5)
Age [years], mean ± SD	3.2 ± 1.3	3.6 ± 1.2	6.7 ± 0.8	8.0 ± 1.6	14.3 ± 2.7	15.8 ± 1.5
Male, *n* (%)	4 (40)	9 (56)	2 (67)	6 (67)	1 (50)	4 (80)
Race, *n* (%)						
Caucasian	9 (90)	16 (100)	3 (100)	8 (89)	2 (100)	5 (100)
Not reported	1 (10)	0	0	1 (11)	0	0
Ethnicity, *n* (%)						
Hispanic or Latino	1 (10)	1 (6)	0	1 (11)	0	2 (40)
Not Hispanic or Latino	8 (80)	15 (94)	3 (100)	7 (78)	2 (100)	3 (60)
Not reported	1 (10)	0	0	1 (11)	0	0
Tumor type, *n* (%)						
Lymphoma	1 (10)	0	0	0	0	2 (40)
Neuroblastoma	6 (60)	12 (75)	1 (33)	2 (22)	0	0
Sarcoma	0	0	2 (67)	6 (67)	2 (100)	2 (40)
Medulloblastoma	1 (10)	2 (13)	0	1 (11)	0	0
Wilms’ tumor	1 (10)	1 (6)	0	0	0	0
Anaplastic ependymoma	1 (10)	0	0	0	0	0
Malignant rhaboid tumor	0	1 (6)	0	0	0	0
Intracranial germ cell tumor	0	0	0	0	0	1 (20)

Patient percentages are a proportion of the treatment arm for all age groups combined (standard mobilization group or plerixafor + standard mobilization group).

**Table 5:** Number (%) of patients with PB CD34+ cell count (cells/µL) less than 10, 15, or 20 on the day before first apheresis.

	Standard arm (*N* = 15)	Plerixafor arm (*N* = 30)
Central laboratory measurement, *n* (%)		
<10 cells/µL	3 (20)	4 (13.3)
<15 cells/µL	4 (26.7)	14 (46.7)
<20 cells/µL	4 (26.7)	17 (56.7)

**Table 6:** Summary of stage 2 efficacy endpoints^a^.

Primary endpoint	Standard arm (*N* = 15)	Plerixafor arm (*N* = 30)
Primary analysis		
Successful mobilization^b^, *n/N*1(%), [95% CI]	4/14 (28.6) [8.4%, 58.1%]	24/30 (80.0) [61.4%, 92.3%]
Difference of proportion of successful mobilization^c^	51.4	
95% CL	[18.5%, 84.3%]	
* p* value^d^	0.0019	

Secondary and exploratory endpoints	Standard arm (*N* = 15)	Plerixafor arm (*N* = 30)
Number of days required to reach ≥2 × 10^6^ cells/kg, median [95% CI]	1 [NC, NC]	1 [NC, NC]
Cumulative patients reaching ≥2 × 10^6^ cells/kg, *n* (%)^a^		
Day 1	15 (100)	26 (89.7)
Day 2	15 (100)	27 (94.4)
Day 3	15 (100)	27 (94.4)
Day 4	15 (100)	27 (94.4)
Day 5	15 (100)	27 (94.4)
Patients not reaching target of ≥2 × 10^6^ cells/kg by apheresis day 5, *n* (%)	1 (7.1)	3 (10.3)
Total blood volume processed [L], median	3.27	3.00
Cumulative CD34+ cell collection (10^6^ cells/kg)^e^		
Number	14	29
Median	10.2	9.1
Min:Max	0.7:66.0	0.1:200.4
Number of patients who received transplants	10 (66.7)	23 (76.7)
Number of CD34+ cells (10^6^ cells/kg) infused on transplant day, median (min:max)	4.55 (0.5:9.4)	4.16 (0.1:12.0)
Percentage of durable engraftment after transplant, *n/n* (%), [95% CI]^f^		
Month 3	10/10 (100) [69.2, 100.0]	21/23 (91.3) [72.0, 98.9]
Month 6	9/10 (90.0) [55.5, 99.7]	20/23 (87.0) [64.4, 97.2]
Month 12	8/10 (80.0) [44.4, 97.5]	20/23 (87.0) [64.4, 97.2]
Month 24	8/10 (80.0) [44.4, 97.5]	19/23 (82.6) [61.2, 95.0]
Time to neutrophil engraftment [days], Median (95% CI)^g^	14 (11.0%, 15.0%)	12 (11.0%, 13.0%)
Time to platelet engraftment [days], Median (95% CI)^g^	23 (11.0%, 31.0%)	28 (18.0%, 37.0%)
Peripheral Blood CD34+ cell count (cells/μL) summary, median (min:max)		
Day prior to first planned apheresis	35.0 (5.0:300.00)	15.0 (1.0:306.0)
Day 1 first planned apheresis	64.0 (11.00:510.00)	77 (0.0:959.0)
Day 1 increase in CD34+ counts from day prior	12 (−17.0:362.0)	53.0 (−45.0:653.0)
Reason for study discontinuation, *n* (%)	Standard arm (*N* = 15)	Plerixafor arm (*N* = 30)
Progressive disease	1 (6.7)	0
Death^h^	3 (20.0)	3 (10.0)
Investigator’s choice^i^	0	1 (3.3)
Subject/parent request	1 (6.7)	1 (3.3)

*CI* confidence interval, *n* number of patients who successfully mobilized, *N*1 number of patients excluding patients missing both local and central laboratory records, *NC* not calculated.

^a^Data for the primary analysis is from the central laboratory. In case of missing data from the central laboratory, the corresponding local laboratory result was used for primary analysis.

^b^The primary endpoint of successful mobilization is defined as at least a doubling of the peripheral blood (PB) CD34+ count from the morning of the day preceding the first planned apheresis day to the morning prior to apheresis. The 95% CIs are calculated using the exact method.

^c^The difference in the proportion of patients who met the primary endpoint is calculated relative to standard mobilization alone treatment arm. The CI of the difference is based on the Wald asymptotic CI with continuity correction method.

^d^The *p* value is based on the Fisher’s exact test comparing the proportion of successful mobilization between the two treatment arms.

^e^Data from central laboratory.

^f^CI is estimated using exact method. Percentages are based on *N*1.

^g^CL estimated by Kaplan–Meier method using log–log transformation.

^h^Death due to progressive disease.

^i^Investigator felt the patient needed more mobilization after first apheresis.

### Efficacy

More patients in the plerixafor arm (24/30, 80%) met the primary endpoint of doubling the PB CD34+ cell count in the 24 h prior to first apheresis than in the standard arm (4/14, 28.6%) (*p* = 0.0019; Table 6). Three patients in the plerixafor arm and one patient in the standard arm failed to reach 2 × 10^6^ CD34+ cells/kg (Table 6). The median total CD34+ cell yield was numerically higher in the standard arm (Table 6).

During the 6-month time period following mobilization 23/30 (76.7%) patients in the plerixafor arm and 10/15 (66.7%) patients in the standard arm proceeded to HSCT. All patients who received HSCT had successful engraftments. The median number of CD34+ cells infused at transplant was 4.55 × 10^6^ cells/kg in the standard arm and 4.16 × 10^6^ cells/kg in the plerixafor arm (Table 6). Rates of neutrophil, platelet, and sustained engraftment were comparable between treatment arms (Table 6).

On the day prior to the first planned apheresis median PB CD34+ cell counts were lower in the plerixafor arm than in the standard arm; by the day of apheresis median PB CD34+ cell counts were higher in the plerixafor arm than in the standard arm (Table 6). An ad hoc analysis demonstrated that patients in the plerixafor arm (*n* = 27) experienced a median PB CD34+ cell increase of 3.2-fold in the 24 h prior to first apheresis compared with a 1.39-fold increase for patients in the standard arm (*n* = 14).

### Stage 2 safety results

In the plerixafor arm 76.7% of patients experienced a TEAE compared with 66.7% of patients in the standard arm (Table 7). The number of TEAEs grade 3 or higher were comparable between arms (43.3% plerixafor arm vs. 40.0% standard arm) (Table 7). No TEAEs grade 3 or higher or SAEs related to study treatment were observed. TEAEs assessed as related to the study treatment were reported for four patients (13.3%) in the plerixafor arm and no patients in the standard arm: injection site reactions (6.7%), hypokalemia (3.3%), blood bicarbonate increased (3.3%). No patient discontinued study treatment due to a TEAE or experienced a TEAE leading to death. The number of patients with myeloablative conditioning before transplantation are shown in Table 8 (stage 1 and stage 2). Six patients (three in each study arm) died during the study; all deaths were due to disease progression and occurred during the posttreatment follow-up period.

**Table 7:** Overview of treatment-emergent adverse events during stage 2.

Parameter, *n* (%)	Standard arm (*N* = 15)	Plerixafor arm (*N* = 30)
Any TEAE	10 (66.7)	23 (76.7)
TEAE related to study procedure	4 (26.7)	12 (40.0)
TEAE related to study treatment^a^	0	4 (13.3)
Injection site reactions		2 (6.7)
Hypokalemia		1 (3.3)
Blood bicarbonate increased		1 (3.3)
Any grade 3–4 TEAE	6 (40.0)	13 (43.3)
Any grade 3–4 TEAE related to study procedure	4 (26.7)	9 (30)
Any grade 3–4 TEAE related to study treatment	0	0
Any serious TEAE	4 (26.7)	9 (30)
Serious TEAE related to study procedure	1 (6.7)	3 (10)
Serious TEAE related to study treatment	0	0

*TEAE* treatment-emergent adverse event.

^a^All were common terminology criteria for adverse events grade 1.

**Table 8:** Patients with myeloablative conditioning before transplantation (a) stage 1 and (b) stage 2.

(a) Stage 1			
	Age 2–<6 years (*n* = 9)	Age 6–<12 years (*n* = 9)	Age 12–<18 years (*n* = 9)
Patients, *n* (%)	8 (88.9)	6 (66.7)	5 (55.6)

(b) Stage 2						
	Age group 1–<6 years		Age group 6–<12 years		Age group 12–<18 years	
	Standard arm (*n* = 10)	Plerixafor arm (*n* = 16)	Standard arm (*n* = 3)	Plerixafor arm (*n* = 9)	Standard arm (*n* = 2)	Plerixafor arm (*n* = 5)
Patients, *n* (%)	7 (70)	13 (81)	2 (67)	8 (89)	1 (50)	3 (60)

## Discussion

This study is the first to address the dose and timing of plerixafor in pediatric patients with cancer and allowed for efficacy and safety to be assessed in this patient population.

Stage 1 of this study was conducted to establish the PK of plerixafor in the pediatric population. No apparent dose-dependent effect of plerixafor on CD34+ cumulative cell yield or fold increases were observed observed and efficacy and safety of plerixafor in this study was similar to that observed in adults at all three doses explored. Therefore the recommended adult dose of 240 µg/kg, administered 8–12 h prior to apheresis, was recommended in pediatric patients.

The primary endpoint, defined as at least a doubling of PB CD34+ cell count in the 24 h prior to first apheresis (i.e., prior to first administration of plerixafor in the experimental arm), was significantly higher in the plerixafor arm than in the standard arm (*p* = 0.0019). The primary endpoint allowed for the measure of the role of plerixafor in addition to standard mobilization with G-CSF alone or in combination with chemotherapy in order to not modify the standard mobilization regimens currently used. Of note, on the day prior to the first planned apheresis, CD34+ cell counts were more than twofold higher in patients randomized to the standard arm than in the plerixafor arm. The added value of plerixafor treatment can be seen in the increase in CD34+ cell counts on day 1 of apheresis from the day prior to apheresis, with patients in the plerixafor arm experiencing a greater median fold change (3.2-fold vs. 1.39-fold) in CD34+ cell counts than those in the standard arm.

In adults, plerixafor has been observed to be advantageous in patients considered to be poor mobilizers using the current consensus threshold for poor mobilization in adults (<20 CD34+ cells/µL) [5–7]. However, the adult “consensus” threshold for poor mobilization may not be appropriate in the pediatric setting [8]. Few studies have been published exploring poor mobilization in pediatric patients and therefore there is no current consensus threshold for poor mobilization in this patient group. In a single retrospective study of pediatric patients, who received a mobilization regime consisting of G-CSF for 4 days, prior to collection of CD34+ cells, [8] no differences were observed in the numbers of patients reaching the target cell dose (2 × 10^6^ cells/µL) for autologous PB progenitor cell transplantation in patients with baseline PB CD34+ cells counts of >20 cells/µL compared with those with baseline CD34+ cells counts of 11–20 cells/µL [8]. Of 26 pediatric patients with a PB CD34+ cell count of ≤10 cell/µL, 18 underwent hematopoietic progenitor cell collection, two reached the target CD34+ cell dose after one apheresis, and 16 failed to reach the target [8].

Data for use of plerixafor for mobilization of HSC in pediatric patients are sparse. Studies, with limited numbers of pediatric patients who had failed earlier mobilization with chemotherapy and G-CSF, or G-CSF alone, have demonstrated that plerixafor combined with G-CSF can result in successful mobilization [9–12]. In a more comprehensive study of 33 pediatric patients who had previously failed to mobilize using G-CSF mobilization regimes, 31 patients successfully mobilized CD34+ cells after treatment with plerixafor, with 27 patients meeting the target of 2 × 10^6^ cell/kg after one apheresis procedure and 24 patients proceeded to transplant [13].

In the current study, more patients in the plerixafor arm than in the standard arm successfully mobilized cells it is important to note that both arms had high levels of successful mobilization, suggesting plerixafor may have a specific role in the population of patients who are poor mobilizers [14]. Again, it was noted that the plerixafor arm had a higher proportion of potential poor mobilizers (as defined using the current consensus threshold for poor mobilization in adults, <20 CD34+ cells/µL), than in the standard arm. The higher number of poor mobilizers in the plerixafor group may have contributed to a trend in slower platelet engraftment observed in the plerixafor group, median time to engraftment 28 days in the plerixafor arm compared with 23 days in the standard arm. However, in another study in children with malignant tumors, the median time to platelet engraftment with plerixafor was 16 days (range 9–30 days) [13], which is similar to the median time of 18–21 days for platelet engraftment observed for both plerixafor plus G-CSF and the G-CSF alone arms in adult patients with non-Hodgkin’s lymphoma and MM [15, 16]. It is known that there are differences in the cellular composition of apheresis products recovered from healthy adults who received either plerixafor plus G-CSF or G-CSF alone [17]. Nevertheless, there is no evidence from studies in adult lymphoma or MM patients that potential differences in apheresis product affects platelet engraftment [15, 16]. The results of the previous pediatric published literature, in which high levels of successful mobilization were observed in patients that had previously failed to mobilize on alternative mobilization regimes, provide further support for the use of plerixafor in HSC mobilization [9–13].

Adverse events reported as related to study treatment in the current study were mild and consistent with the known safety profile of plerixafor. In addition, few AEs have been reported in other studies investigating the use of plerixafor in pediatric patients [9, 11, 12]. Maschan et al. [13] noted mild toxicity in 8 out of 33 patients including World Health Organization grade 1/2 diarrhea (*n* = 5), grade 2 nausea (*n* = 2), grade 1 bone pain (*n* = 1), and urticaria (*n* = 1). Hong et al. reported that two patients with medulloblastoma developed pneumomediastinum, with pathogenic findings consistent with diffuse alveolar damage [10].

In summary, this study has established the dose and timing of plerixafor in pediatric patients as 240 µg/kg to be administered between 6 and 11 h before apheresis. More patients in the plerixafor arm, than in the standard arm, met the primary endpoint of successful mobilization supporting the role of plerixafor on increasing the number of mobilized CD34+ cells in PB, especially in poor mobilizers. Adverse events reported as related to study treatment were mild, and no new safety concerns were identified in this study. Overall, plerixafor was generally well tolerated and efficacious when used to mobilize CD34+ cells in pediatric patients with a variety of different cancers.

## Supplementary information

Supplementary Figures

Supplementary Figures Legend

## Data Availability

Qualified researchers may request access to patient-level data and related study documents including the clinical study report, study protocol with any amendments, blank case report form, statistical analysis plan, and dataset specifications. Patient-level data will be anonymized and study documents will be redacted to protect the privacy of trial participants. Further details on Sanofi’s data sharing criteria, eligible studies, and process for requesting access can be found at https://www.clinicalstudydatarequest.com.
