# Supplementary figures and images for: Plerixafor combined with standard regimens for hematopoietic stem cell mobilization in pediatric patients with solid tumors eligible for autologous transplants: two-arm phase I/II study (MOZAIC)
Source: Bone Marrow Transplant. 2020 Mar 3;55(9):1744–53. doi: 10.1038/s41409-020-0836-2 (PMC7452813; doi:10.1038/s41409-020-0836-2)

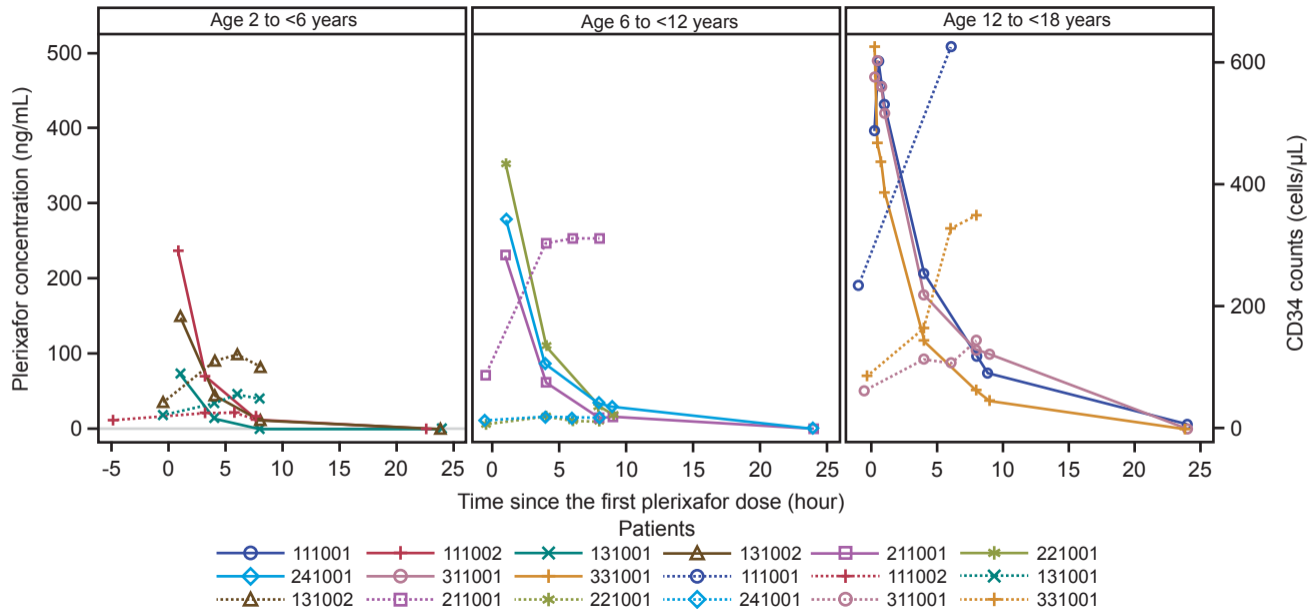

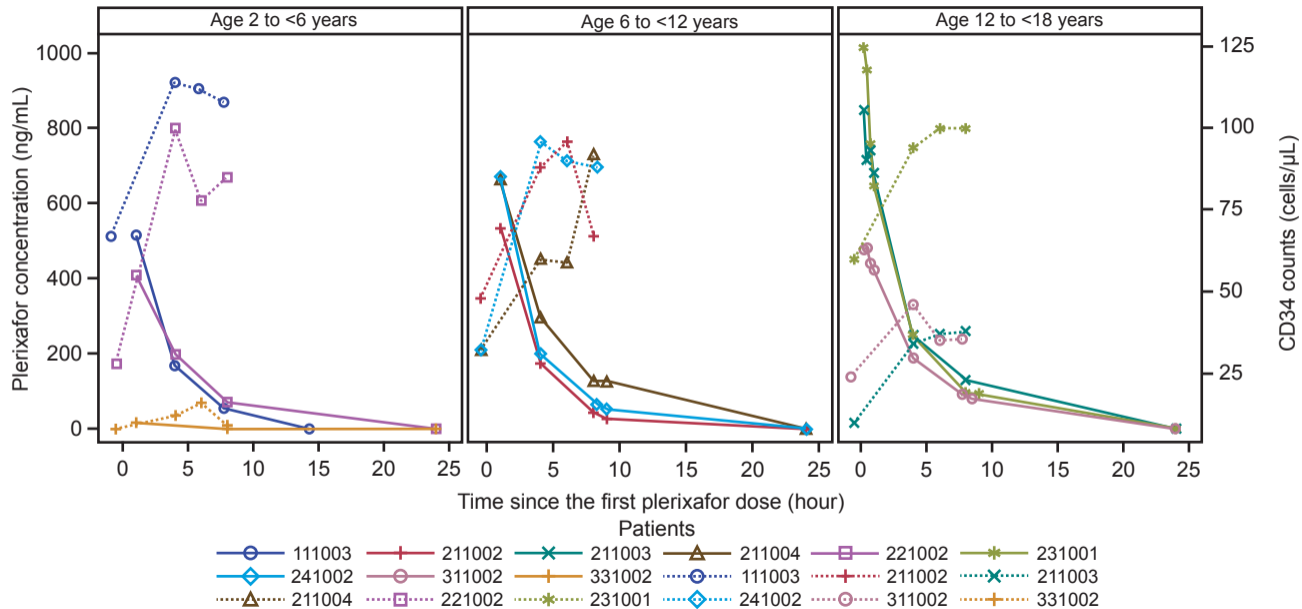

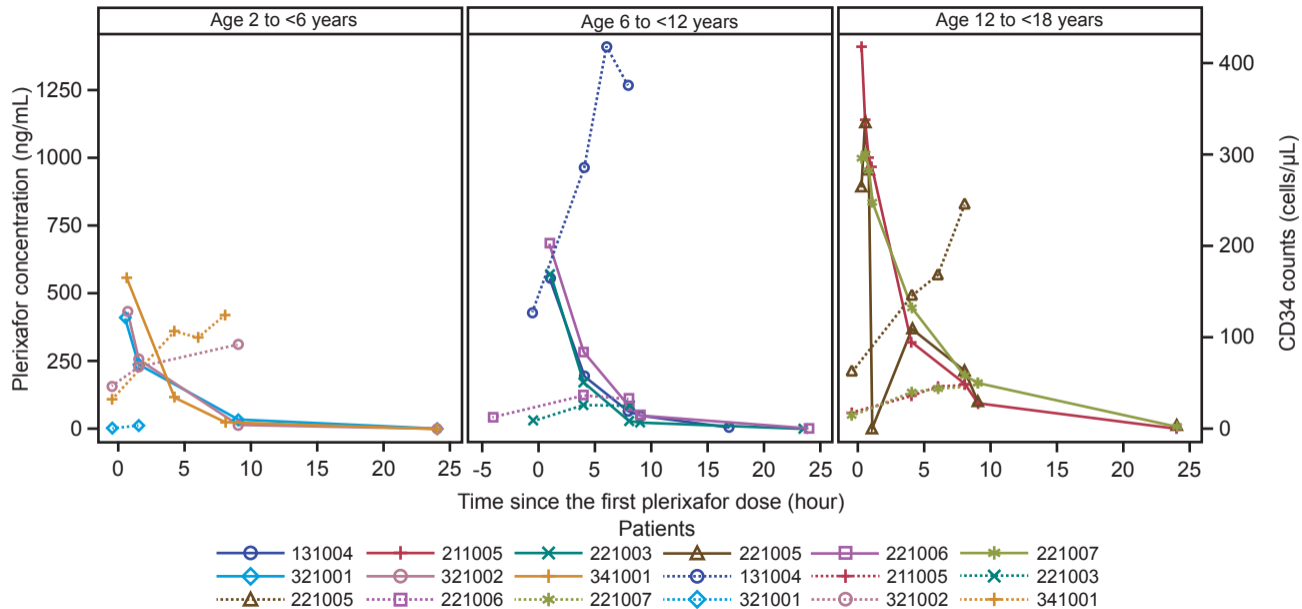

Supplement: Supplementary file 1 — Supplementary Figures [file 41409_2020_836_MOESM1_ESM.pdf]
