# Supplementary material for: Plerixafor combined with standard regimens for hematopoietic stem cell mobilization in pediatric patients with solid tumors eligible for autologous transplants: two-arm phase I/II study (MOZAIC)
Source: Bone Marrow Transplant. 2020 Mar 3;55(9):1744–53. doi: 10.1038/s41409-020-0836-2 (PMC7452813; doi:10.1038/s41409-020-0836-2)
Supplement: Supplementary file 2 — Supplementary Figures Legend [file 41409_2020_836_MOESM2_ESM.docx]

**Supplementary Figure 1. Individual plerixafor concentrations for the 3 age cohorts and PB CD34+ levels at different times after first plerixafor dose administration in the a) 160 μg/kg, b) 240 μg/kg and c) 320 μg/kg groups**

Solid lines represent plerixafor concentration (ng/mL) and dotted lines represent CD34 counts (cells/uL).
Central laboratory data has been used. However, when central lab value is missing, local value is used.
